# Mandatory national language requirements in higher education

**DOI:** 10.1038/s44319-024-00211-9

**Published:** 2024-07-18

**Authors:** Shina Caroline Lynn Kamerlin

**Affiliations:** Georgia Institute of Technology, Atlanta, GA USA

**Keywords:** Careers, Science Policy & Publishing

## Abstract

Some countries try to curb internationalisation in academia or require that foreign scientists take language courses. While this is done for the wrong reasons, learning the language of the host country has great benefits for academic staff.

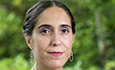

Science is a global enterprise, which requires a common language. The current *lingua franca* of science is English, although this was not always the case: in the past, Arabic, Latin and Greek among others have all fulfilled this role at one point of time or another, and, until just over a century ago, scientific communication was dominated by German and French.

The shift towards English as the dominant language for science during the past century has significantly impacted the higher education landscape in Europe. Where once University courses in non-English speaking countries would have been entirely or mostly in the local language, there are an increasing number of institutions that offer courses or even degree programs in English. There are also pan-European education schemes involving multiple institutions: one example with which I am personally familiar due to my discipline is the Erasmus Mundus Master of Science in Theoretical Chemistry and Computational Modeling, which is taught across seven institutions and entirely in English. Courses in English facilitate international mobility for both students, staff and faculty, and, more broadly, contribute to institutional internationalization, all of which I consider important to higher education as well as for increasing competitiveness in a global economy.

However, recent years have witnessed a rise in far-right politics in Europe with associated nationalistic sentiment, and one of the targets of this sentiment has been the prevalence of English at universities and the ensuing demand for preserving national languages in higher education. An example of this is the Netherlands, where universities have agreed to both limit the number of English language courses, and to curb internationalization. More recently, and against the advice of the Norwegian Directorate for Higher Education and Skills, the Norwegian government—which is most definitely *not* far right—has announced a plan that would require foreign PhD students and postdoctoral fellows to complete 15 mandatory credits in Norwegian during their employment period. To rationalize this policy, the Research and Higher Education Minister Oddmund Hoel stated “We are taking the necessary steps to preserve Norwegian as a professional language and to prevent English from becoming the main language in Norwegian higher education” (translated from Norwegian).

Unsurprisingly, these plans have sparked alarm, in particular among academics, with legitimate concerns about the willingness of foreigners to come to a country if the government is putting up additional barriers for them. While a lot of this push towards mandatory language courses and reduction of teaching in English is part of a broader push against internationalization and nationalistic tendencies across Europe, an important question is whether there may actually be benefits to national language training or requirements for doctoral, postdoctoral and more senior members of academic staff and faculty. I want to note though that I am personally a strong proponent of internationalization in higher education and of teaching courses primarily in English to facilitate such internationalization. Further, my focus on Europe in this piece is for illustrative purposes.

Language training is obviously not an issue that many people think about if they are working in an English-speaking country, since many academics either have English as their first language, or are already proficient in English to the point of being bilingual. Since they already work in an English-speaking environment, there are no issues with integration into local society, and with interacting with the broad range of staff that comprise an institute of higher education.

However, the majority of European countries are non-English speaking, and while the local community may be extremely proficient in English, this cannot be taken for granted. It is worth pointing out though that both the Netherlands and Norway are countries with very high levels of English proficiency. Being able to speak the local language, even just enough to get by, can be useful to be able to integrate more broadly with the local community as well as dealing with practical legal and administrative matters. I speak several languages fluently and have lived in several countries and there have been many situations when even if the person I was communicating with was perfectly proficient in English, the interaction was much more productive when I could speak their language.

Second, a lot of these language discussions have focused on undergraduate courses, or in the case of the Norwegian proposition appear to affect primarily doctoral and postdoctoral researchers. However, higher education at these levels still operates primarily in English in many internationalized European settings. The challenge is that as you move up the seniority ladder, conversations and meetings where important strategic decisions are made—for instance, departmental board meetings, faculty meetings, institutional-level leadership meetings – will increasingly be in the local language. Not being able to speak it can act as a glass ceiling limiting how high foreigners can move into leadership roles, even if their teaching load is mainly in English.

Furthermore and often left out of such discussions are communication with university administrative and support staff—for instance HR, the finance team, facilities management, and the myriad of other important non-academic staff that keep a university running. These people are not necessarily bilingually proficient in English, and even when they are, being able to communicate with them in the local language can be extremely helpful.

Many if not most universities do offer courses in the local language: as some examples, I was able to find Swedish courses at Uppsala University, German courses at the University of Vienna, and French courses at the Sorbonne University in Paris. Clearly, taking advantage of language courses is a good idea, and institutions should increase availability and make them accessible to international faculty and staff. The challenge though is one that plagues all academics, regardless of seniority: finding time amidst the many urgent requirements of our jobs. In this sense, mandatory language training both acts as a motivating factor to complete such courses, but also to remove the time penalty to some extent—when doing these courses you are not expected to do other things. In parallel, we should retain our internationalization efforts, expect university leadership to be proficient in English, and help to integrate international researchers into our university systems.

In summary, while these language propositions may be put forward for potentially—in my opinion—all the ‘wrong’ reasons, there is a strong benefit to gain from learning the local language for international researchers—if implemented in a constructive way that does not put an additional burden on them and enables them to engage more directly with their new host country.

## Supplementary information

Peer Review File

