# Supplementary material for: Mandatory national language requirements in higher education
Source: EMBO Rep. 2024 Jul 18;25(9):3739–40. doi: 10.1038/s44319-024-00211-9 (PMC11387601; doi:10.1038/s44319-024-00211-9)
Supplement: Supplementary file 1 — Peer Review File [file 44319_2024_211_MOESM1_ESM.pdf]

EMBO reports

Mandatory National Language Requirements in Higher Education

Shina Caroline Lynn Kamerlin

Corresponding author(s): Shina Caroline Lynn Kamerlin (skamerlin3@gatech.edu)

Review Timeline:

Submission Date:	11th Jul 24
Accepted:	11th Jul 24

Editor: Holger Breithaupt

Transaction Report: This article type is not peer reviewed.
